# Taurine Promotes Differentiation and Maturation of Neural Stem/Progenitor Cells from the Subventricular Zone via Activation of GABA_A_ Receptors

**DOI:** 10.1007/s11064-023-03883-2

**Published:** 2023-03-02

**Authors:** Nadia Estefanía Gutiérrez-Castañeda, Jessica González-Corona, Ernesto Griego, Emilio J. Galván, Lenin David Ochoa-de la Paz

**Affiliations:** 1 Laboratorio de Neurobiología Molecular y Celular de la Glía, Unidad de Investigación UNAM-APEC, 04030 México City, México; 2 Departamento de Farmacobiología, Centro de Investigación y de Estudios Avanzados del Instituto Politécnico Nacional, 14330 México City, México

**Keywords:** Taurine, GABA Receptors, Subventricular Cells, Stem/progenitor Cell, Neurogenesis, Differentiation, Morphogenesis

## Abstract

Neurogenesis, the formation of new neurons in the brain, occurs throughout the lifespan in the subgranular zone of the dentate gyrus and subventricular zone (SVZ) lining the lateral ventricles of the mammal brain. In this process, gamma-aminobutyric acid (GABA) and its ionotropic receptor, the GABA_A_ receptor (GABA_A_R), play a critical role in the proliferation, differentiation, and migration process of neural stem/progenitor cells (NPC). Taurine, a non-essential amino acid widely distributed throughout the central nervous system, increases the proliferation of SVZ progenitor cells by a mechanism that may involve GABA_A_R activation. Therefore, we characterized the effects of taurine on the differentiation process of NPC expressing GABA_A_R. Preincubation of NPC-SVZ with taurine increased microtubule-stabilizing proteins assessed with the doublecortin assay. Taurine, like GABA, stimulated a neuronal-like morphology of NPC-SVZ and increased the number and length of primary, secondary, and tertiary neurites compared with control NPC of the SVZ. Furthermore, neurite outgrowth was prevented when simultaneously incubating cells with taurine or GABA and the GABA_A_R blocker, picrotoxin. Patch-clamp recordings revealed a series of modifications in the NPCs’ passive and active electrophysiological properties exposed to taurine, including regenerative spikes with kinetic properties similar to the action potentials of functional neurons.

**Supplementary Information:**

The online version contains supplementary material available at 10.1007/s11064-023-03883-2.

## Introduction

Neurogenesis, the process of generating functional neurons from precursor cells, was traditionally associated with mammals’ embryonic and perinatal developmental stages [1]. However, the milestone discovery by Altman and Das (1965) [2] demonstrating the presence of newborn neurons in the adult brain showed for the first time that active neurogenesis continues throughout the lifespan of the mammalian brain [2]. Today, active adult neurogenesis is acknowledged as a neurodevelopmental process that includes cell proliferation, migration, differentiation, cell fate decision, and functional integration into pre-existing neuronal networks [3]. Adult neurogenesis is a dynamic and accurate process regulated by multiple physiological, pharmacological, and pathological stimuli [4]. Previous studies demonstrated that pharmacological modulation of gamma-aminobutyric acid (GABA) transmission regulates neurogenesis in the adult brain [5, 6], suggesting that GABA-activated responses trigger a series of molecular and biochemical mechanisms relevant to the neurogenesis process.

Under physiological conditions, neurogenesis is restricted to two neurogenic regions: the subgranular zone of the dentate gyrus, an area that generates new granule cells [2]; and the subventricular zone (SVZ) of the lateral ventricles, where new neurons are generated and migrate through the rostral migratory stream to the olfactory bulb where they mature into interneurons [7].

It is well documented that GABA has a depolarizing activity in neural precursor cells during brain development, controlling cell division and contributing to neuronal migration and maturation. Moreover, neural stem/progenitor cells (NPC) express at least GABA_A_ and metabotropic GABA receptors (GABA_B_R) in the subventricular and subgranular zones [8]; in these cells, GABA causes depolarization and affects proliferation, migration, and neuronal maturation [9]. Electrophysiological studies have documented that the tonic release of GABA activates GABA_A_ receptors (GABA_A_R) expressed in neuroblasts and stem cells [10–13]. In addition, GABA, or the selective GABA_A_R agonist, muscimol, activates a chloride current in neonatal NPC-SVZ [10]. In line with these findings, acute treatment of type B and C SVZ cells with muscimol decreases cell proliferation, assessed with a bromodeoxyuridine incorporation assay [14]. Interestingly, taurine, a neuroactive molecule and structurally related analog of GABA, mimics the GABA action by activating GABA_A_R [15, 16].

Taurine (2-aminoethane sulfonic acid) is an abundant free amino acid found in the mammalian brain [17] and is also present in the heart, retina, and muscle tissues [18]. Humans obtain taurine directly from meat consumption, as a secondary source, or via a reduced biochemical synthesis process [19]. Taurine is synthesized from cysteine, in which cysteine dioxygenase and cysteine sulfinic acid decarboxylase form hypotaurine. Hypotaurine is converted by the hypotaurine dehydrogenase enzyme to taurine [20]. Experimental evidence demonstrates that taurine acts as a trophic factor, stimulates proliferation, and promotes neurogenesis of NPC-SVZ cultures [21]. These observations suggest that taurine plays a central role in the process of proliferation, differentiation, and migration of progenitor cells during the embryonic development of the central nervous system. However, the mechanism by which taurine participates in NPC differentiation of the SVZ is unclear. Because taurine is one of the most abundant free neuroactive molecules from an early CNS developmental stage and GABA_A_R is present on SVZ progenitor cells, the present study aimed to evaluate the effect of taurine on the differentiation process of NPC-SVZ through interaction with GABA_A_R.

## Materials and Methods

### Cell Culture

All the animals were handled following the guidelines of the National Institutes of Health Guide for Care and Use of Laboratory Animals and with the approval of the Institutional Animal Care and Use Committee of the National University of Mexico (protocol # 068-2019/018-CIC-2019). Only 8-day-old CD1 mice were used to obtain NPC-SVZ, in total 100 animals were used for 4 experimental groups (25 animals per group). Animals were collected from a laboratory animal facility and decapitated as soon as they arrived at the laboratory, the brain was removed and the SVZ was isolated. No randomization was performed to allocate subjects in the study and no blinding was performed. The tissue obtained was mechanically dissociated, and placed in DMEM/F12 (Dulbecco’s Modified Eagle Medium/Nutrient Mixture F-12. Gibco; Thermo Fisher Scientific, Inc., Waltham, MA, USA) medium, and centrifuged for 5 min at 1000 rpm. The supernatant was removed, and the button was resuspended and cultured in a growth medium (DMEM/F12 supplemented with B27, plus epidermal growth factor and fibroblast growth factor 2) to promote progenitor cells proliferation and SVZ-neurosphere formation. The culture was kept at 37 °C and 5% CO_2_. After 7 days, primary neurospheres were disaggregated with trypsin for 5 min, and a trypsin inhibitor (1:1) was added to stop the reaction. Once the cells were separated, they were recultured in a fresh growth medium for 4–5 days at 37 °C and 5% CO_2_. For the differentiation process of NPC-SVZ, disaggregated cells of the secondary SVZ-neurospheres were cultured in 12 wells pretreated with poly-D-lysine (Sigma-Aldrich, St. Louis, MO, USA), and differentiation medium (DMEM/F12 supplemented with 1% fetal bovine serum [FBS]) and complemented with taurine (10 mM) or GABA (100 µM). A dose-response was performed to determine the effective concentration for taurine, cell cultures were exposed to taurine at several concentrations (from 2.5 to 60 mM). To evaluate the role of GABA_A_R in the differentiation process, cells were preincubated for 1 h with picrotoxin (5 µM, 50 µM, and 100 µM); after this time, the medium was stirred to withdraw the stimulus and a differentiation medium or treatment with taurine (10 mM) or GABA (100 µM) was applied. GABA was used as a positive control to compare the mechanism of taurine on NPC differentiation processes.

## Immunofluorescence Assay

Cells were fixed with cold 4% paraformaldehyde, washed with phosphate-buffered saline (PBS) + 0.1% bovine serum albumin (BSA; 3 × 5 min each), permeabilized/blocked with PBS + 0.1% BSA + 10% FBS + 0.3% Triton X-100 for 1 h at room temperature. The cells were subsequently incubated overnight with the primary antibodies nestin (Cell Signaling Technology Cat# 4760, RRID:AB_2235913) [1:300]; glial fibrillary acidic protein (GFAP; Cell Signaling Technology Cat# 12,389, RRID:AB_2631098) [1:100]; and doublecortin (DCX; Santa Cruz Biotechnology Cat# sc-271,390, RRID:AB_10610966) [1:100]. The primary antibodies were removed the following day, followed by 1-h incubation with the secondary antibodies Alexa Fluor-488 anti-rabbit 9 for GFAP and SOX-2) and Alexa Fluor-647 anti-mouse (for nestin and DCX). Next, the excess secondary antibodies were washed and removed from the preparation assembly. The nuclei were stained with Hoechst (20 µg/mL) included in the mounting solution (100/500 µL).

### Quantitative Analysis for cell Markers

Microphotographs were obtained with the cell imaging multi-mode plate reader Cytation™ 5 (BioTek Instruments Inc., Winooski, VT, USA) using a green fluorescent protein (GFP) filter cube (excitation 469/35 nm; emission 525/39 nm; dichroic mirror 497 nm) and 465 nm LED; Texas Red filter cube (excitation 586/15 nm; emission 647/67 nm; dichroic mirror 605 nm) and 590 nm LED; and 4′,6-diamidino-2-phenylindole (DAPI) filter cube (excitation 377/50 nm; emission 447/60 nm; dichroic mirror 409) and 365 nm LED. Microphotographs of five different fields were randomly selected from each coverslip, and the number of totals (Hoescht positive) cells and cells positive for the different markers in the selected fields were quantified. Cell images were processed using Gen5™ software (BioTek Instruments Inc.). The results were expressed as the percentage of positive cells of the total cell number.

### Morphometric Analysis of DCX Positive Cells

Microphotographs of DCX positive (DCX+) cells were randomly obtained with a 20x magnification lens. DCX + cells were analyzed for each experimental condition (control, taurine, and GABA). The number of primary, secondary, and tertiary neurites and the length of the primary neurites were determined with Gen 5 3.04. The difference between primary, secondary, and tertiary neurites was analyzed in the different experimental conditions. Primary neurites were defined as those that originated directly from the soma; secondary and tertiary neurites were defined as those that originated from primary and secondary neurites, respectively [22].

### Whole-cell Patch-clamp Recordings

Dissociated NPC-SVZ from SVZ-neurospheres were taken from a petri dish containing cell culture, mounted on a coverslip, and transferred to a submersion recording chamber. They were visualized with infrared differential interference contrast optics coupled to a Nikon FN1 microscope (Nikon Corporation, Minato, Tokyo, Japan). The NPC-SVZ were continuously perfused with Ringer’s solution at the rate of 3–4 mL.min^− 1^ with the help of a peristaltic pump (120 S, Watson-Marlow, Wilmington, MA, USA). The composition of Ringer’s solution was the following (mM): 125 NaCl, 2.5 KCl, 1.25 Na_2_HPO_4_, 25 NaHCO_3_, 2 MgCl_2_, 2 CaCl_2_, and 10 D-glucose; pH  7.30–7.40; osmolarity = 280–290 mOsm. The recording chamber temperature was maintained at 32 ± 1 °C with a single channel temperature controller (TC-324 C, Warner Instruments, Hamden, CT, USA). For the whole-cell patch-clamp recordings of hippocampal interneurons included in this study, the parameters were published elsewhere [23]. The patch pipettes were pulled from borosilicate glasses using a micropipette puller (P57, Sutter Instruments, Novato, CA, USA). The puller was programmed to obtain pipettes with a tip resistance of 5–7 MΩ when filled with an intracellular solution containing (mM): 135 K^+^-gluconate, 10 KCl, 5 NaCl, 1 EGTA, 10 HEPES, 2 Mg^2+^-ATP, 0.4 Na^+^-GTP, 10 phosphocreatine, and pH 7.20–7.30. Whole-cell recordings were performed with an Axopatch 200B amplifier (Molecular Devices, San José, CA, USA). Acquired data were digitized at a sampling frequency of 10 kHz and filtered at 5 kHz with a Digidata 1322 A (Axon Instruments, Palo Alto, CA, USA). Signals were acquired and analyzed offline with pCLAMP 10.6 software (Molecular Devices).

### Determination of Intrinsic Properties

The resting membrane potential (RMP) was determined after the initial break-in from giga-seal to whole-cell configuration. Once the cell was switched to current-clamp mode, a series of negative and positive current steps from − 300 to rheobase (1 s, 30 pA increments) were injected to determine input resistance (R_N_) and membrane time constant (τ_memb_). R_N_ was calculated as the slope value of a first-order polynomial function *f*(*x*) = *mx* + *b* fitted to the current-voltage relationship near RMP. τ_memb_ was determined by fitting a mono-exponential decay function to a voltage response elicited by injecting a negative current step (1 s, –30 pA). Membrane capacitance (C_m_) was calculated as the ratio of τ_memb_ to R_N_, as previously reported [24].

### Determination of Firing Output Properties

We analyzed the firing properties of recorded NPC-SVZ by constructing current-frequency plots. For these measurements, the firing frequency was plotted against the intensity of the injected current. Experimental data were fitted to a three-parameter logistic function.

### Spike and Action Potential Analyses

The spike threshold was calculated using the third derivative’s first peak intersecting with the spike onset. The spike kinetic properties were analyzed by constructing phase plots. In this analysis, the first derivative of the action potential (AP) / (mV.ms^−1^) was plotted against the membrane potential, as previously reported [25].

### Statistical Analysis

No statistical method was used to determine the sample size. The sample size was arbitrarily set to 100 (4 groups with 25 animals each). Statistical analysis was performed with GraphPad Prism 6. The graphs represent the mean ± standard error of the mean. Data normality was previously determined with a Kolmogorov-Smirnov test. Most of the data did not meet the normality criteria, so non-parametric tests were applied: Mann-Whitney U for comparing two groups and Kruskal-Wallis for comparing more than two groups. A one-way analysis of variance (ANOVA) test was applied, followed by Tukey’s *post hoc* test in cases where normal distribution was observed. In cases where the comparison was between two groups, the Student’s t-test was applied. In all cases, the accepted level of significance was *P* < 0.05.

## Results

### Characterization of NPC Markers in SVZ-derived Neurospheres

Cultured SVZ-neurospheres are widely used to mimic NPC’s proliferative capacity, self-renewal, and differentiation into mature cell lineages; therefore, we used SVZ neurospheres to characterize the effects of taurine on the differentiation processes of NPC-SVZ. The neurospheres were obtained from 8-day-old CD1 mice and cultured in a growth medium with two passages (see *Materials and Methods* for details). Primary, secondary, and tertiary SVZ-neurospheres were formed at 7 ± 1, 12 ± 1, and 17 ± 1 days, respectively (Suppl Fig. 1A–C). For this study, all the experimental manipulations were restricted to secondary SVZ-neurospheres (Suppl Fig. 1B) to minimize biased interpretations given the cellular changes that occur during self-renewal (unorganized structure) or senescence (necrotic center) of SVZ-neurospheres [26].

We first determined the presence of progenitor and neuronal markers in the secondary SVZ-neurospheres. Immunofluorescence image analysis revealed nestin and SOX-2 immunopositivity (Suppl Fig. 2A, B); contrary to this observation, we did not detect DCX immunofluorescence (Suppl Fig. 2C). The immunopositivity to SOX-2 and nestin confirms the progenitor origin of the NPC-SVZ, whereas the lack of DCX immunopositivity indicates a lack of differentiation to immature neurons.

### Expression of Neuronal Markers in NPC-SVZ Exposed to Taurine or GABA

**Fig. 1:**
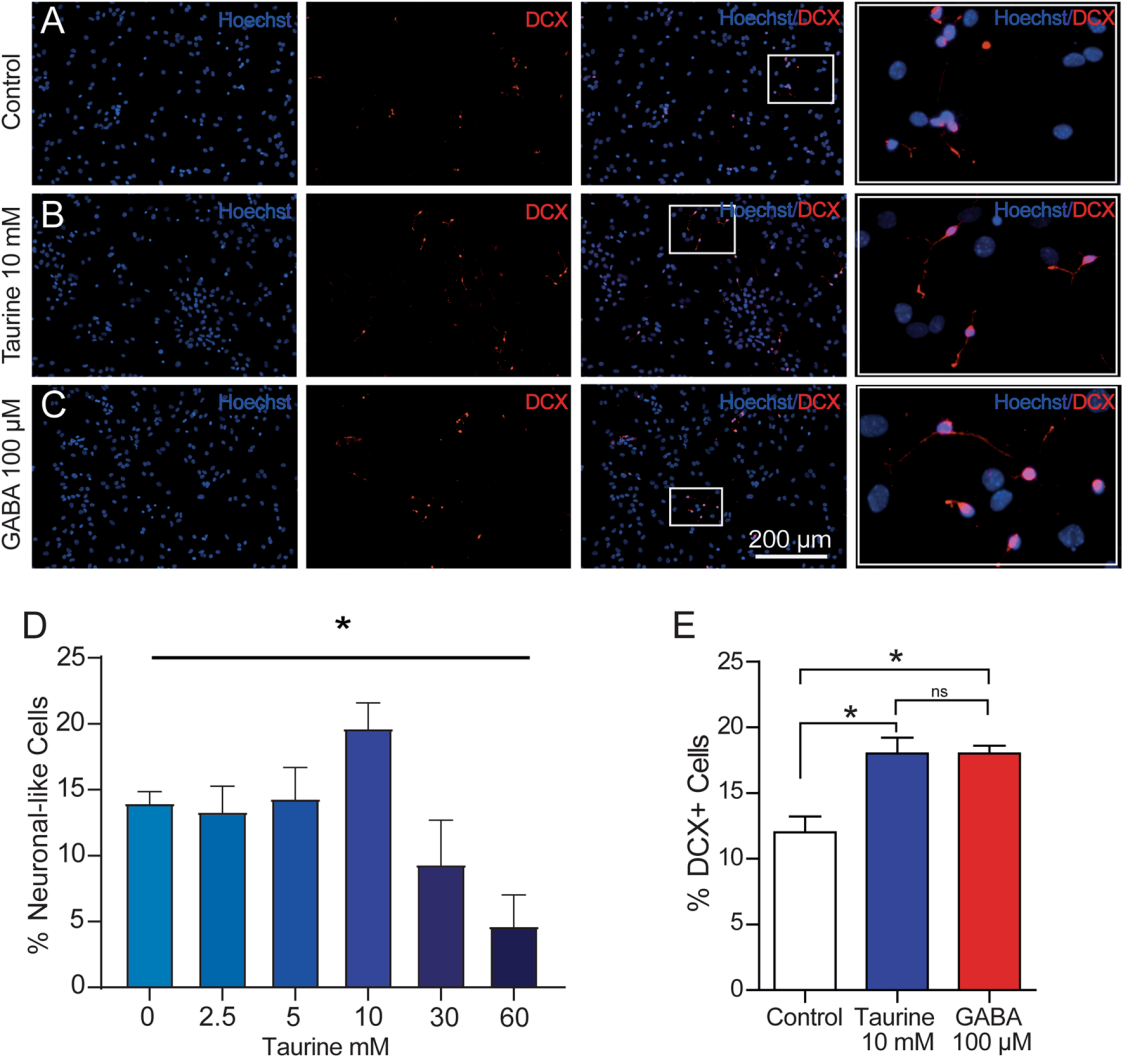
Immunolabeling for neuronal markers in disaggregated NPC-SVZ Immunopositivity of disaggregated cells to DCX was observed consistently in **A** control cells, **B** cells treated with 10 mM taurine, and **C** cells treated with 100 µM GABA (positive control). The nuclei were stained with Hoescht (blue labeling). Right panels are magnifications obtained from the white boxes in the preceding panels showing the neurite outgrowths in the three experimental conditions. **D** Dose-response relationship with taurine, observe that 10 mM taurine was the effective concentration to generate neuron-like cells. **E **Boxplot graph showing a normalized count of DCX + cells in each experimental condition. Treatment with taurine or GABA significantly increases the number of DCX + cells. *P < 0.05

Next, the expression of the neuronal markers during the differentiation process was analyzed in disaggregated cells. Because previous studies have documented the expression of neuronal markers in the presence of GABA [27], we used this pharmacological condition as a positive control. Therefore, cells were exposed to taurine (10 mM) or GABA (100 µM) for 7 days, and the immunocytochemical analyses were performed against DCX, a standard marker to identify the neuronal identity of differentiated cells [28]. Compared with control cells (Fig. 1A), the cultures exposed to taurine or GABA (Fig. 1B, C, respectively) exhibited increased immunopositivity to DCX (one-way ANOVA; *P* < 0.05), with no statistical difference in DCX expression between the treated groups (Fig. 1E). Moreover, the cells exposed to taurine exhibited a clear morphological difference compared with control cells: small and elongated or multipolar somata with cytoplasmic outgrowths or neurites with a neuronal-like morphology (Fig. 1B; right panel). Although cells exposed to GABA also exhibited neurites, these structures were less conspicuous than those observed with taurine (Fig. 1C; right panel). The dose-response relationship showed a significant increase in the rate of DCX + cells exposed to taurine at 10 mM (Fig. 1D). These results suggest that taurine stimulates the expression of neuronal markers and has stronger induction properties than GABA to generate neurites in disaggregated NPC-SVZ.

### Taurine Induces neuronal-like Characteristics in NPC-SVZ

**Fig. 2:**
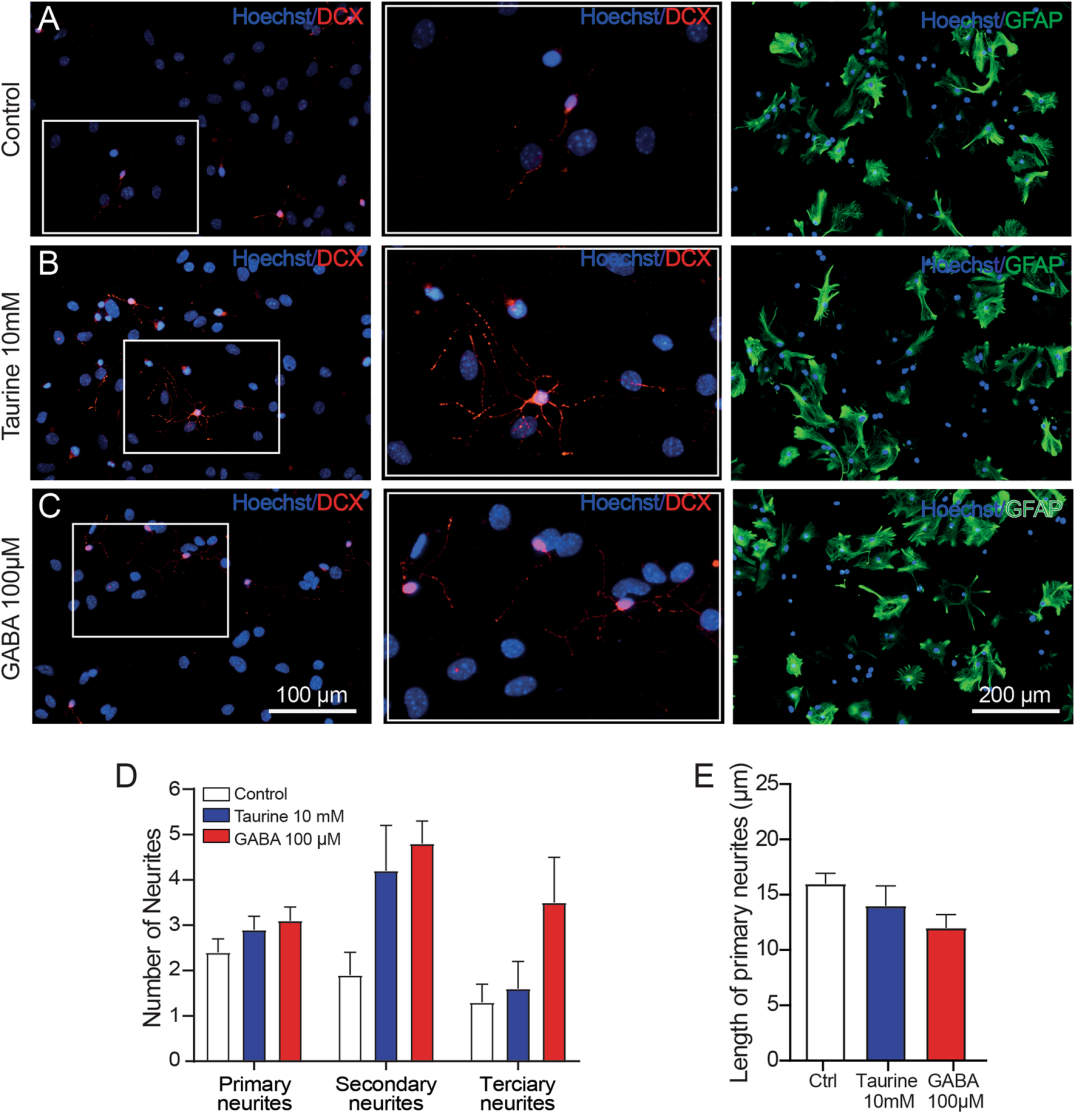
Neurite outgrowth is restricted to DCX + and absent in GFAP + cells. **A**–**C** Left panels, disaggregated NPC-SVZ showing immunopositivity to DCX. Middle panels: corresponding magnified microphotographs from the left panels. Treatment with taurine or GABA stimulated the formation of neurites. Right panels: cells positive to GFAP lacked neurite outgrowths. **D** Bar graph showing the average number of neurite outgrowths measured from somata. **E** Bar graph showing the average length of primary neurites in the three experimental conditions

Because the NPC-SVZ can differentiate to both glial and neuronal cells [29], we next determined if taurine or GABA influences the differentiation of the disaggregated NPC-SVZ to glia. Compared with control cells (Fig. 2B), treatment with taurine (10 mM) or GABA (100 µM; Fig. 2B, C) promoted somatic elongation and neurite outgrowth that conferred a neuronal-like morphology of the treated cells. However, this morphology was restricted to DCX + cells (middle panels in Fig. 2B, C); more importantly, it was absent in cells immunopositive to GFAP (right panels in Fig. 2A–C; see also Suppl. Figure 3). The DCX + cells with neurite outgrowths exhibited rounded somata, had a length of 52.07 ± 4.8 μm, and a nuclei size of 12.18 ± 1.05 μm. By contrast, the GFAP + cells exhibited several somatic shapes, including elongated, stellate, or round morphology with abundant cytoplasm (133.3 ± 9,21 μm) and elongated nuclei (14.05 ± 0.8 μm) (right panels in Fig. 2A and Suppl. Fig. 3).

A morphometric analysis of DCX + cells revealed differences in the differentiation process in each experimental condition. For instance, cells treated with taurine or GABA exhibited numerous secondary and tertiary neurites compared with control cells. The number of primary, secondary, and tertiary neurites in each experimental condition is summarized in the bar graph in Fig. 2D (one-way ANOVA; *P* > 0.05). Regarding the length of primary neurites, we did not find a statistical difference in the three experimental conditions (Fig. 2E).

**Fig. 3:**
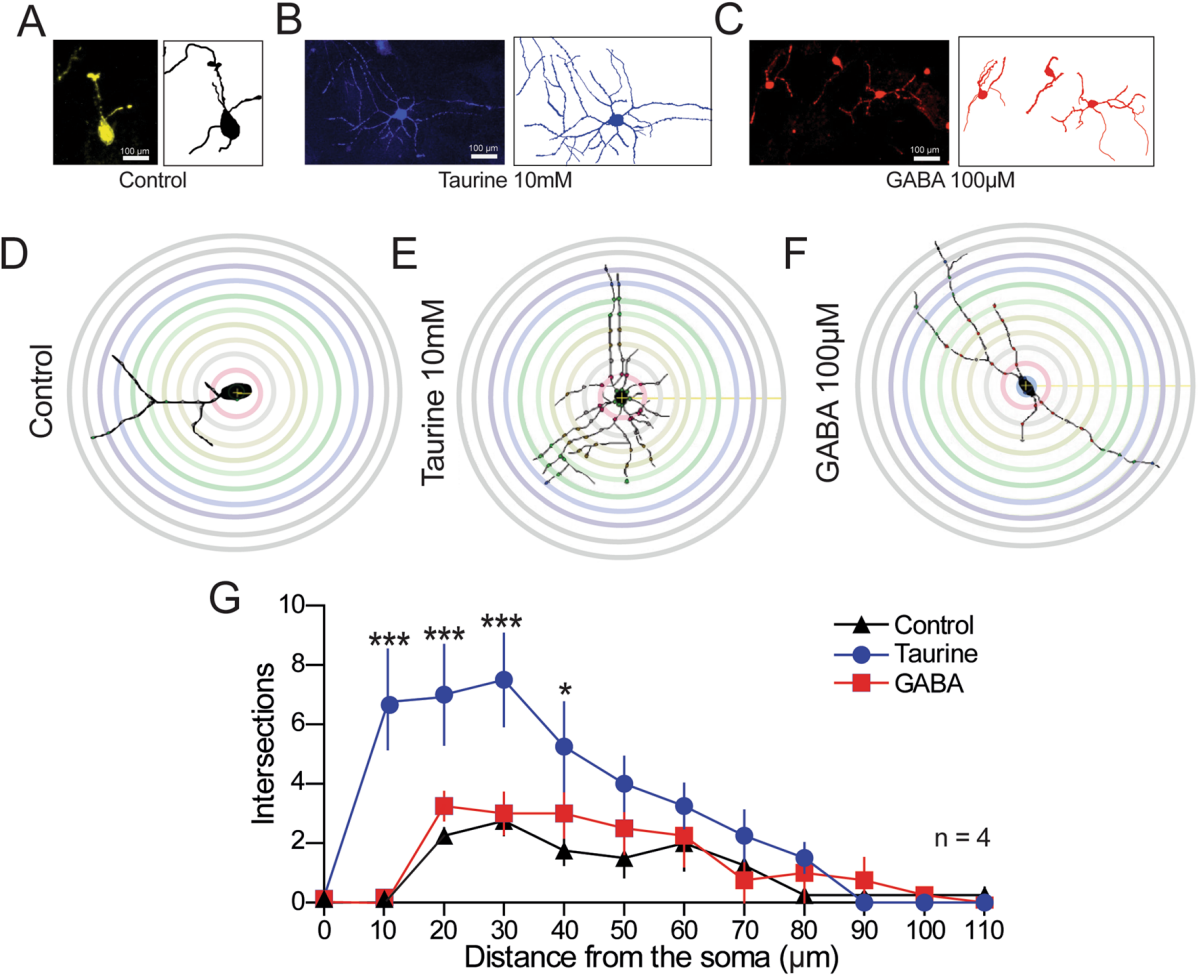
Representation of the Sholl analysis. **A**–**C** Microphotographs of representative NPC-SVZ in the three experimental conditions showing the typical neurite outgrowth and their corresponding digital reconstructions. **D**–**F** Schematic representation of the morphometric Sholl analysis performed on control, taurine- and GABA-treated cells. **G** The number of intersections between neurite outgrows and concentric rings (10 μm increments) from the center of the soma were used to determine primary, secondary, and tertiary ramifications (n = 5). *P < 0.05; ***P < 0.001

We performed a Sholl analysis to achieve more accurate morphometry of the differentiated cells. Representative microphotographs of cells from the three experimental conditions and their corresponding digital reconstructions are depicted in Fig. 3A–C. Concentric rings superimposed on the digital reconstructions for the Sholl analysis are depicted in Fig. 3D–F. Control cells exhibited unipolar morphology with fewer neurites (Fig. 3A, D). In sharp contrast, cells treated with taurine exhibited multipolar morphology and a robust number of neurites and arborizations (Fig. 3B, E). Furthermore, cells treated with GABA exhibited a bipolar-like morphology, with a reduced number of neurites and no arborizations compared with those observed in taurine-treated cells. (Fig. 3 C, F). The number of neurite intersections is plotted against neurite length from the soma, thus summarizing neuronal complexity, in Fig. 3G. As expected, the number of neurite intersections was larger in cells treated with taurine than those expressed in control cells or from cells treated with GABA. These morphometric analyses strongly suggest that taurine acts as a strong promoter of neurite complexity and contributes to the neuronal maturation of differentiated NPC.

### Blockade of GABA_A_R Suppress the Morphogenic Process Induced by Taurine

**Fig. 4:**
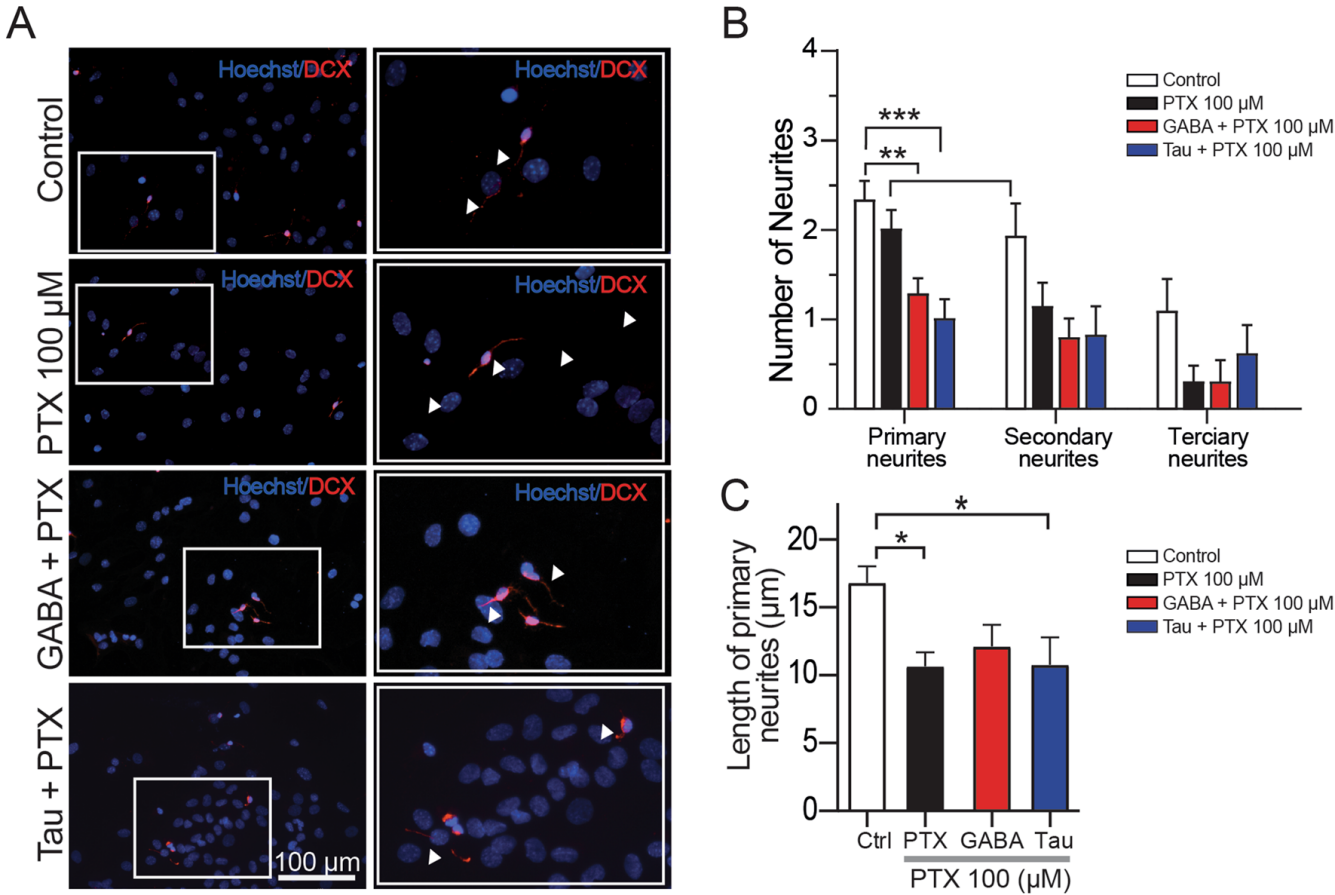
Effect of PTX on the differentiation and maturation process. **A** Left panels, disaggregated NPC-SVZ immunopositive to DCX; the cells were incubated with 100 µM PTX. Right panels, corresponding magnifications showing the reduced number of neurites in the presence of PTX. Arrowheads indicate neurite outgrowth. **B** Bar graph showing the average number of primary, secondary, and tertiary neurite outgrowths measured from the somata. **C** Bar graph contrasting the average length of primary neurites in control and the three experimental conditions. *P < 0.05; **P < 0.01; ***P < 0.001

Previous studies demonstrated that GABA_A_R participates in the morphogenic process of NPC-SVZ [5, 30]. To investigate if GABA_A_R are involved in the morphogenic process mediated by taurine, disaggregated NPC-SVZ were differentiated in increasing concentrations of the GABA_A_R antagonist, PTX (5, 50, and 100 µM, respectively). We first corroborated that preincubation with the increasing doses of PTX did not induce cell damage. For this, a cell counting kit-8 assay determined that none of the PTX concentrations caused significant cell damage (data not shown). These observations also showed that 100 µM PTX caused a considerable reduction of neurites in cells exposed to taurine or GABA. Therefore, the number and length of neurites were analyzed in DCX + cells exposed to 100 µM PTX. Differentiated cells were preincubated with 100 µM PTX for 1 h. Under these conditions, there was a significant decrease in the number of primary neurites in DCX + cells treated with either GABA plus PTX (one-way ANOVA; *P* < 0.01; middle panel, Fig. 4A and red bars in B) or taurine plus PTX (one-way ANOVA; *P* < 0.001; bottom panel, Fig. 4A and blue bars in B). We also determined that blockade of GABA_A_R with 100 µM PTX reduced the number of secondary and tertiary neurites (one-way ANOVA; *P* > 0.05; Fig. 6B).

Likewise, the length of primary neurites exhibited a significant decrease in the presence of PTX alone or combined with taurine or GABA (one-way ANOVA; *P* < 0.05 for all comparisons; Fig. 6C). These experiments demonstrate for the first time that activation of GABA_A_R is a necessary step in the taurine-mediated morphogenic process and neurite outgrowth of NPC-SVZ.

### Taurine or GABA Treatment Induces Electrophysiological Responses in Disaggregated NPC

**Fig. 5:**
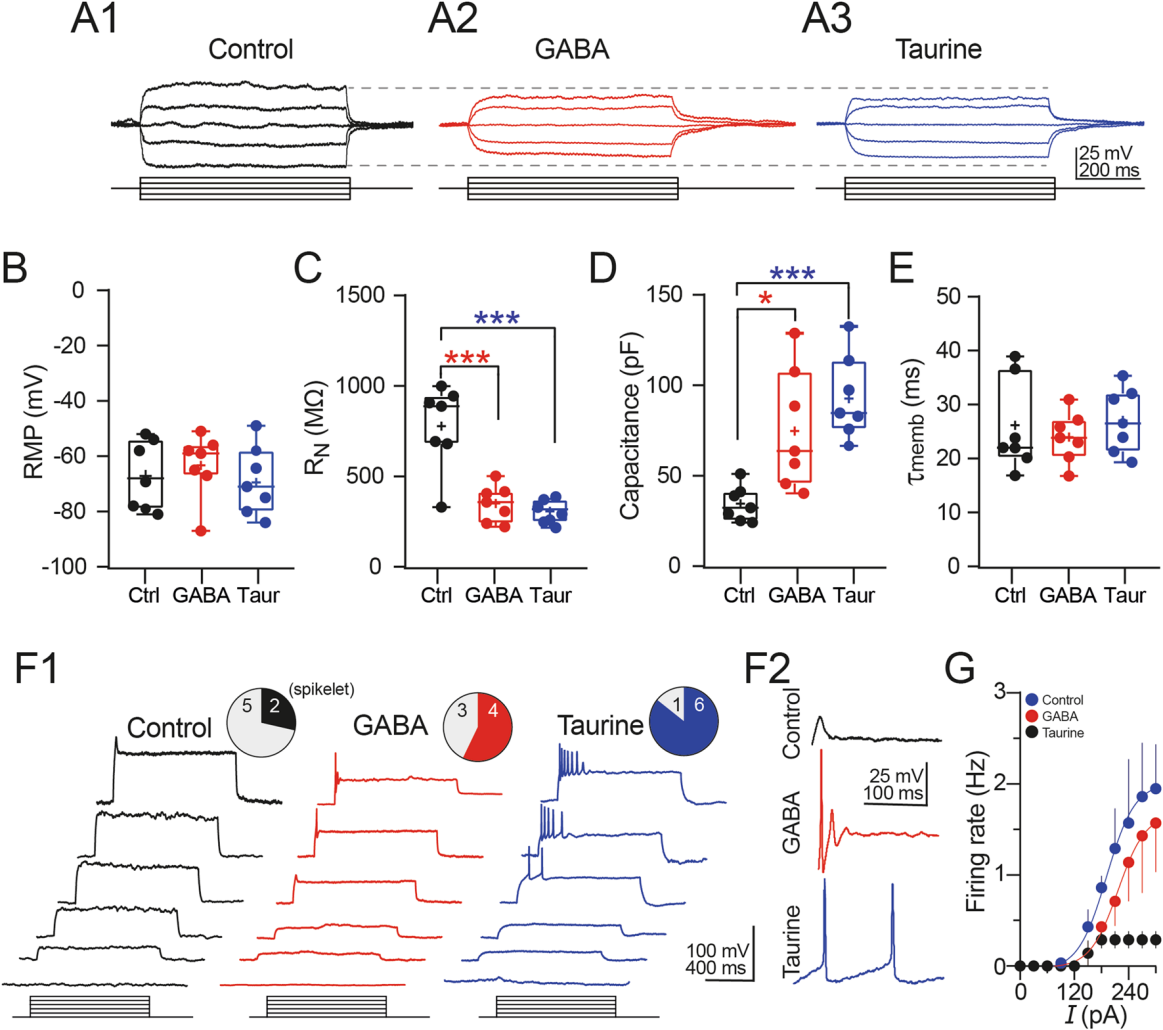
Electrophysiological properties of disaggregated NPC-SVZ treated with taurine or GABA. **A1**–**3** Representative current-voltage responses elicited at − 60 mV from control, GABA, and taurine-treated cells. Note the decreased voltage response of cells treated with GABA or taurine (continuous dashed lines). Box plots and individual experiments (circles) showing: **B** RMP, **C** somatic R_N_, **D** somatic C_m_, and **E** t_memb_ of control, GABA-, and taurine-treated cells. The solid line in the boxplots represents the median and the cross represents the mean. **F1** Representative voltage responses elicited at -60 mV. Increasing depolarization triggered a ‘spikelet’ in control cells (left panels); in GABA- (middle panel), and taurine-treated cells (right panel), a brief burst of spikes were observed. The upper pie charts show the proportion of cells capable of eliciting a spike in response to depolarizing current injection. **F2** Magnification of representative spikes obtained in the three experimental conditions. GABA- and taurine-treated cells exhibited a well-defined AHP conductance following individual AP. **G** Plot of the averaged maximum spike firing frequency response to depolarizing current steps for each experimental condition. *P < 0.05; ***P < 0.01 or higher statistical significance; n = 7 disaggregated cells for each experimental condition

Because of our previous finding that cells differentiated with taurine or GABA express neuronal markers and exhibit a neuronal-like morphology, we next determined the passive and active electrophysiological properties of the NPC-SVZ with whole-cell patch-clamp recordings. We measured membrane properties from 21 cells divided into control, taurine- and GABA-treated cells (n = 7 for each group). Figure 5A1–3 show representative voltage responses to current injections obtained under the three experimental conditions. Compared with control cells, the decreased voltage deflections observed in GABA- or taurine-treated cells (see the continuous horizontal dash lines) indicate increased functionality of ion channels active near the RMP, possibly voltage-dependent and inward-rectifying potassium channels. From the current-voltage relationships, we determined that neither GABA nor taurine altered the RMP compared with control cells (RMP in: control cells = -67.1 ± 4.7 mV; GABA-treated cells = –63.3 ± 4.4 mV; taurine treated cells = –69.4 ± 4.6 mV; one-way ANOVA; F_(2,18)_ = 0.456; *P* = 0.641; Fig. 5B). Consistent with the decreased voltage deflections as a result of increased ion channel activity near the RMP, we observed a marked decrease in the somatic R_N_ of NPC-SVZ treated with GABA or taurine compared with control cells (R_N_ in: control cells = 777.7 ± 87.66 MΩ; GABA-treated cells = 350.1 ± 38.2 MΩ [Tukey’s test; *P* < 0.001]; taurine-treated cells = 307.3 ± 22.55 MΩ [Tukey’s test; *P* < 0.001]; one-way ANOVA; F_(2,18)_ = 21.040; *P* < 0.001; Fig. 5C). The changes in R_N_ were accompanied with an increase in the cell’s C_m_ (C_m_ in: control cells = 34.6 ± 6.7 pF; GABA-treated cells = 75.9 ± 12.6 pF [Tukey’s test; *P* = 0.012]; taurine-treated cells = 92.7 ± 8.7 pF [Tukey’s test; *P* < 0.001]; one-way ANOVA; F_(2,18)_ = 10.860; *P* < 0.001; Fig. 5D). Furthermore, the C_m_was higher in cells treated with taurine than those treated with GABA, consistent with the cell’s volume regulation being mediated by taurine [31, 32]. Lastly, we did not find differences in t_memb_ between the three experimental conditions (τ_memb_ in: control cells = 25.7 ± 3.21 ms; GABA-treated cells = 23.9 ± 1.74 ms; taurine-treated cells = 27.8 ± 2.25 ms; one-way ANOVA; F_(2,18)_ = 0.616; *P* = 0.550; Fig. 5E).

**Fig. 6:**
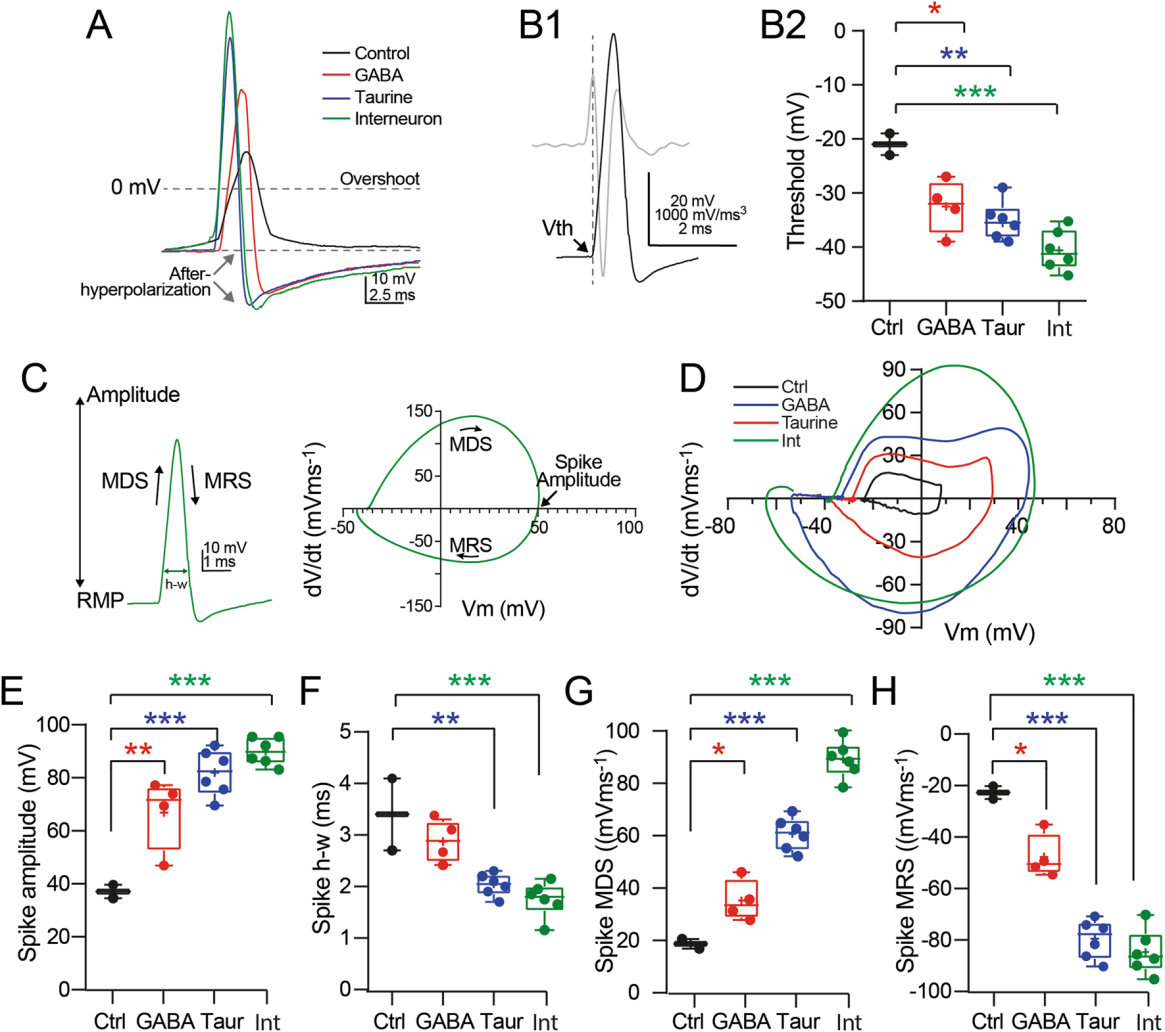
Kinetic properties of NCP-SVZ spikes. **A** Superimposed spikes from control, GABA-, and taurine-treated cells. The green trace corresponds to an actual AP from a hippocampal interneuron recorded from an acute hippocampal slice. Note the control spike’s slow onset, reduced overshoot, and lack of AHP. In contrast, GABA and taurine treatment increased the spike’s onset, overshoot, and repolarization. **B1** Representative examples of the first and third derivatives (grey and black trace, respectively) of an actual AP or spike. The V_th_ for spike initiation was determined by the third derivative changes from negative to positive. **B2** Box plots and individual experiments showing Vth for each experimental group, including the values obtained from an actual AP (green box and symbols). GABA and taurine treatment shifted V_th_ to a more hyperpolarized membrane potential. **C**) Example of an actual AP (from a hippocampal interneuron) and the resulting phase plot indicating the kinetic parameters measured. MDS = maximum depolarization slope; MRS = maximum repolarization slope; h-w = spike half-width. **D** Superimposed phase plots from control, GABA-, and taurine-treated cells and an actual AP. Box plots and individual experiments (symbols) summarizing the kinetic parameters obtained from spikes in each experimental condition. GABA and taurine treatment: **E** increased spike amplitude; **F** decreased spike h-w, **G** increased spike MDS, and **H** decreased MRS. *P < 0.05; **P < 0.01; ***P < 0.01; n = 7 disaggregated cells for each experimental condition

Next, we explored the active membrane properties of disaggregated NPC-SVZ. Increasing current steps were injected to test if a sustained membrane depolarization triggers AP. Control cells did not elicit an AP, as illustrated in Fig. 6F1 (left panels), and only two control cells exhibited a short, voltage-sensitive ‘spikelet’ (upper panel, Fig. 6F1) upon sustained depolarization. On the other hand, cells treated with GABA exhibited an increased proportion of cells that elicited at least one fast spike that resembled an actual AP (middle panel, Fig. 6F1). Contrary to these observations, repetitive AP discharge was observed in the taurine-treated cells (right panel, Fig. 6F1). The number of cells that exhibited AP-like waveforms (solid fill) versus those with no response (grey fill) in the three experimental conditions is summarized in the upper pie charts of Fig. 6F1. The proportion of: control cells eliciting a spikelet = 28.57%; GABA-treated cells eliciting at least one spike = 57.14%; taurine-treated cells eliciting at least one spike = 85.71%).Interestingly, the AP observed in the taurine-treated cells was followed by an afterhyperpolarization (AHP) that resembled the Ca^2+^-dependent slow AHP that follows the AP of central neurons [33]. The firing pattern observed in the three experimental conditions and the resulting AHP is depicted in Fig. 6F2. We also expressed the repetitive firing in terms of AP frequency. Figure 6G summarizes the AP frequency as a function of the injected current of control (black symbols), GABA- (red symbols), and taurine-treated cells (blue symbols). Together, these results support the hypothesis that taurine is a potent inductor of phenotypic differentiation of NPC maintained in vitro and show that taurine is more effective than GABA in transforming NPC-SVZ to a neuron-like phenotype with electrophysiological properties resembling those of central neurons.

### Action Potential Properties of the Differentiated NPC-SVZ

The voltage traces in Fig. 6F2 show that incubation with GABA or taurine reshapes the spike waveform. This effect is more evident in the superimposed, magnified spikes in Fig. 6A. As previously stated, the control ’spikelet’ was smaller and slower, with an amplitude that barely exceeded the AP overshoot, lacked an AHP, and required strong depolarization for initiation. On the other hand, GABA- and taurine-treated cells exhibited fast and regenerative spikes with prominent overshoots followed by an AHP conductance (Fig. 6A). Given these findings, we next analyzed the kinetic properties of the spikes and compared the resulting parameters with averaged values obtained from actual AP spikes from hippocampal interneurons. First, we determined the voltage membrane threshold (V_th_) for spike initiation by computing the third derivative of the spike, a mathematical method that identifies an actual AP threshold [34] (Fig. 6B1). Compared with control cells, we found that both GABA and taurine treatment shifts the spike V_th_ to a more hyperpolarized membrane potential, closer to the V_th_ of an actual AP (spike V_th_ in: control cells = −21.1 ± 2.00 mV; GABA-treated cells = −32.5 ± 2.50 mV [Tukey’s test; *P* = 0.022]; taurine-treated cells = −35.2 ± 1.44 mV [Tukey’s test; *P* = 0.005]; one-way ANOVA; F_(2,9)_ = 9.352; *P* = 0.006; actual AP V_th_ = −42.33 ± 1.54 mV [Tukey’s test; *P* < 0.001]; one-way ANOVA; F_(3,14)_ = 15.840; *P* < 0.001; Fig. 6B2).

Next, we analyzed the kinetic parameters of the spikes utilizing a phase plot analysis. The left panel in Fig. 6C shows an actual AP spike from a hippocampal interneuron, and in the right panel, its resulting geometrical representation or phase plot; the superimposed phase plots from the three experimental conditions plus an actual AP (green trace) are superimposed in Fig. 6D.

Compared with control cells, GABA and taurine treatment increased the spike amplitude (spike amplitude in: control cells = 27.1 ± 2.55 mV; GABA-treated cells = 66.9 ± 6.83 mV [Tukey’s test; *P* = 0.021]; taurine-treated cells = 81.9 ± 3.58 mV [Tukey’s test; *P* = 0.001]; one-way ANOVA; F_(2,9)_ = 14.330; *P* = 0.002; Fig. 6E). NPC-SVZ treated with taurine exhibited an amplitude closer to the actual AP amplitude (actual AP amplitude = 90.97 ± 2.12 mV [Tukey’s test; *P* < 0.001]; one-way ANOVA; F_(3,14)_ = 20.940; *P* < 0.001; Fig. 6E). Consistent with this finding, the spike half-width (h-w) was shorter in the GABA- and taurine-treated cells and in the latter group was closer to the actual AP h-w (spike h-w in: control cells = 3.4 ± 0.70 ms; GABA-treated cells = 2.9 ± 0.20 ms [Tukey’s test; *P* = 0.379]; taurine-treated cells = 2.0 ± 0.09 ms [Tukey’s test; *P* = 0.009]); one-way ANOVA; F_(2,9)_ = 9.198; *P* = 0.007; actual AP h-w = 1.7 ± 0.14 [Tukey’s test; *P* < 0.001]; one-way ANOVA; F_(3, 14)_ = 12.910; *P* < 0.001; Fig. 6F). Likewise, the maximum depolarization slope (MDS) of the spike, a parameter that reflects activity of the AP’s voltage-dependent Na^+^ conductance, was increased in GABA- and taurine-treated cells (MDS in: control cells = 18.7 ± 1.80 mV/ms; GABA-treated cells = 32.8 ± 4.42 mV/ms [Tukey’s test; *P* = 0.049]; taurine-treated cells = 58.1 ± 2.57 mV/ms [Tukey’s test; *P* < 0.001]; one-way ANOVA; F_(2,9)_ = 32.260; *P* < 0.001; actual AP MDS = 88.7 ± 2.88 mV/ms [Tukey’s test; *P* < 0.001]; one-way ANOVA; F_(3, 14)_ = 79.130; *P* < 0.001; Fig. 6G). Lastly, we also computed the maximum repolarization slope (MRS) as an indirect measure of voltage-dependent K^+^ conductances underlying the AP repolarization phase. Compared with control cells, GABA and taurine treatment increased the speed of the MRS (MRS in: control cells = −22.7 ± 2.50 mV/ms; GABA-treated cells = −47.7 ± 4.32 mV/ms [Tukey’s test; *P* = 0.011]; taurine-treated cells = −79.5 ± 3.08 mV/ms [Tukey’s test; *P* < 0.001]; one-way ANOVA; F_(2,9)_ = 48.870; *P* < 0.001; actual AP MRS = −84.7 ± 3.56 mV/ms [Tukey’s test; *P* < 0.001]; one-way ANOVA; F_(3, 14)_ = 42.630; *P* < 0.001; Fig. 6H). Together, these data demonstrate that taurine, like GABA, induces phenotypic plasticity and modulates electrophysiological properties of disaggregated NPC-SVZ. Moreover, our data strongly suggest that taurine has a stronger effect compared with GABA in the differentiation process of NPCs.

## Discussion

In this work, we have provided experimental evidence regarding the multipotentiality of NPC-SVZ to generate cells that express neuronal markers and develop electrophysiological properties following treatment with the non-essential amino acid taurine. In line with this finding, we demonstrated the critical role of the ionotropic GABA_A_R in the differentiation process mediated by taurine. The interaction between the GABA_A_R and taurine promotes a neuron-like phenotype of NPC-SVZ, increases their dendritic complexity, and favors an electrophysiological behavior that resembles actual neuron activity, including the onset of repetitive firing.

We first confirmed that NPC-SVZ matured in neurospheres expressing nestin and SOX-2 markers, as reported by Sanchez-Mendoza (2016) [35]. Moreover, disaggregation of neurospheres to monolayer cultures treated with taurine increased the percentage of cells expressing the neuronal marker DCX, an observation previously reported for GABA [27] and confirmed in this study.

Ramos-Mandujano (2014) [36] proposed that taurine promotes mitochondrial efficiency and increases ATP availability, an immediate effect that increases neuronal proliferation. In this work  we offered data demonstrating both the critical role that the GABA_A_R has in the differentiation process of NPC-SVZ, and that blockade of this receptor suppresses differentiation. In agreement with our findings, the participation of GABA_A_R in the neurogenic process has been previously observed in cortical precursor cells, in which activation of the GABA_A_R triggers a sustained depolarization in postnatal and adult precursor cells, promoting axon elongation, neurite growth, and synaptogenesis [37–39]. Therefore, one possible way to explain the role of GABA_A_R in the taurine-mediated NPC-SVZ differentiation process is that upon their activation, a GABA_A_R-mediated sustained depolarization favors cytosolic calcium accumulation and activates Ca^2+^-dependent mechanisms associated with neurite formation [40]. However, additional experiments are required to confirm this. As previously stated, the critical role of GABA_A_R in the neuronal differentiation of NPC-SVZ was demonstrated when disaggregated NPC-SVZ were simultaneously exposed to taurine and the GABA_A_R blocker PTX. Blockade of GABA_A_R decreased the number of cells immunopositive to DCX; furthermore, these cells exhibited fewer secondary and tertiary neurons. These observations strongly indicate that taurine modulates the differentiation processes of NPC-SVZ to neuronal cells via activation of GABA_A_R. In this sense, it is important to emphasize that modulation of GABA_A_R by taurine may promote other neurogenic regulatory mechanisms, including the decreased activity of glial cells [40]; activation of the sonic hedgehog signaling pathway [41]; increased mitochondrial activity [35, 42], or activation of GABA_B_R.

Our Sholl analyses confirmed that taurine has a stronger differentiation effect than GABA. This observation was confirmed with the morphometric analyses that verified the more complex neurite branching and increased formation of secondary and tertiary neurites in taurine- versus GABA-treated cells. Thus, it can be assumed that taurine participates in the maturation and incorporation of NPC-SVZ to perform neuronal circuits by promoting axonal differentiation, dendrite arborization, and the formation of synaptic connections [37–39].

Another remarkable observation of this study is the emergence of defined electrophysiological properties of disaggregated NPC-SVZ exposed to taurine or GABA. Compared with control cells that barely exhibited neurite outgrowth, cells exposed to GABA, and more importantly taurine, exhibited changes in their passive and active membrane properties. Taurine caused a dramatic drop in the R_N_ and increased the activity of membrane conductances active near the RMP. Moreover, the proportion of cells generating repetitive APs was increased. In line with this observation, taurine-treated cells exhibited a well-defined AHP phase that resembles the Ca^2+^-activated K^+^ conductances that underlie and shape the AP output of mature neurons. Taurine caused NPC-SVZ cells to behave as an actual AP of a mature neuron by altering several kinetic parameters, including AP threshold, spike amplitude, and h-w, as well as AP depolarization and repolarization rates. More importantly, these effects were more marked in cells treated with taurine than GABA, supporting the hypothesis that taurine represents a stronger morphogen than GABA.

## Conclusion

We have provided in vitro evidence regarding the role of taurine as a key neurogenic factor in the differentiation process of NPC-SVZ. We have demonstrated that taurine induces functional neurons by a mechanism that requires the activation of GABA_A_R. The morphological and electrophysiological changes mediated by taurine support the hypothesis that this non-essential sulfur-containing amino acid has functional implications in the neurogenesis process, including gene transcription, neural protein expression, and transport system activity. Given the non-active biochemical nature of taurine, our observations may provide a novel pharmacological tool to renew, increase, or maintain the pools of stem cells that participate in the neurogenesis process during or after a pathological condition, or for the treatment of the age-related reduction in adult neurogenesis.

## Supplementary Information

Below is the link to the electronic supplementary material.

Supplementary file1 (TIF 6966 KB)—Neurospheres obtained from SVZ from a mouse brain. Representative bright-field microphotographs of A) primary, B) secondary, and C) tertiary neurospheres. Microphotographs in the left panels were taken with a 10x magnification lens; right panel, 20x. Neurospheres were formed at 7, 12, and 17 days, respectively

Supplementary file2 (TIF 7172 KB)—Expression of NPC markers in NPC- neurospheres.Representative immunofluorescence microphotographs of secondary neurospheres before differentiation. The nuclei were stained with Hoescht (blue signal). Neurospheres were immunopositive to **A) **nestin and** B)** SOX-2. **C)** Neurospheres did not exhibit immunopositivity for DCX. Microphotographs were taken with a 20x magnification lens. The scale bar applies to all panels

Supplementary file3 (TIF 34018 KB)—Effect of taurine and GABA on the GFAP expression of disaggregated NPC-SVZ. A–C) Representative immunofluorescence microphotographs of disaggregated NPC-SVZ after the differentiation process in the three experimental conditions showing GFAP+ cells, including their characteristic morphology: abundant cytoplasm with elongated, stellate, or round shapes. The nuclei were stained with Hoescht (blue signal). The scale bar applies to all the microphotographs. B) Bar graph showing the percentage of GFAP+ cells in each experimental condition

## Data Availability

The datasets generated during and/or analyzed during the current study are available under request.
