# Supplementary figures and images for: Taurine Promotes Differentiation and Maturation of Neural Stem/Progenitor Cells from the Subventricular Zone via Activation of GABAA Receptors
Source: Neurochem Res. 2023 Mar 2;48(7):2206–19. doi: 10.1007/s11064-023-03883-2 (PMC10181976; doi:10.1007/s11064-023-03883-2)

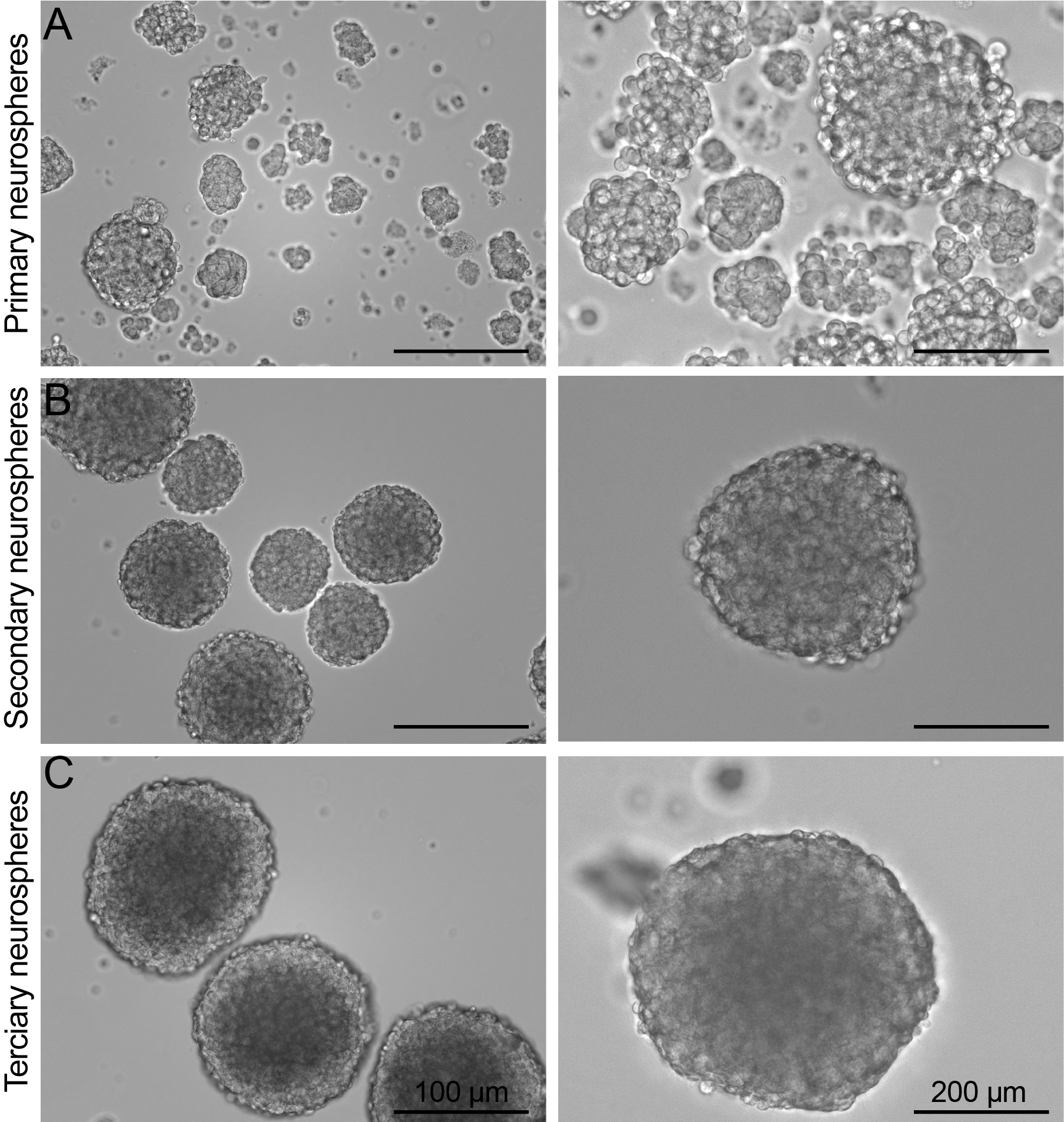

Supplement: Supplementary file 1 — Supplementary file1 (TIF 6966 KB)—Neurospheres obtained from SVZ from a mouse brain. Representative bright-field microphotographs of A) primary, B) secondary, and C) tertiary neurospheres. Microphotographs in the left panels were taken with a 10x magnification lens; right panel, 20x. Neurospheres were formed at 7, 12, and 17 days, respectively [file 11064_2023_3883_MOESM1_ESM.tif]

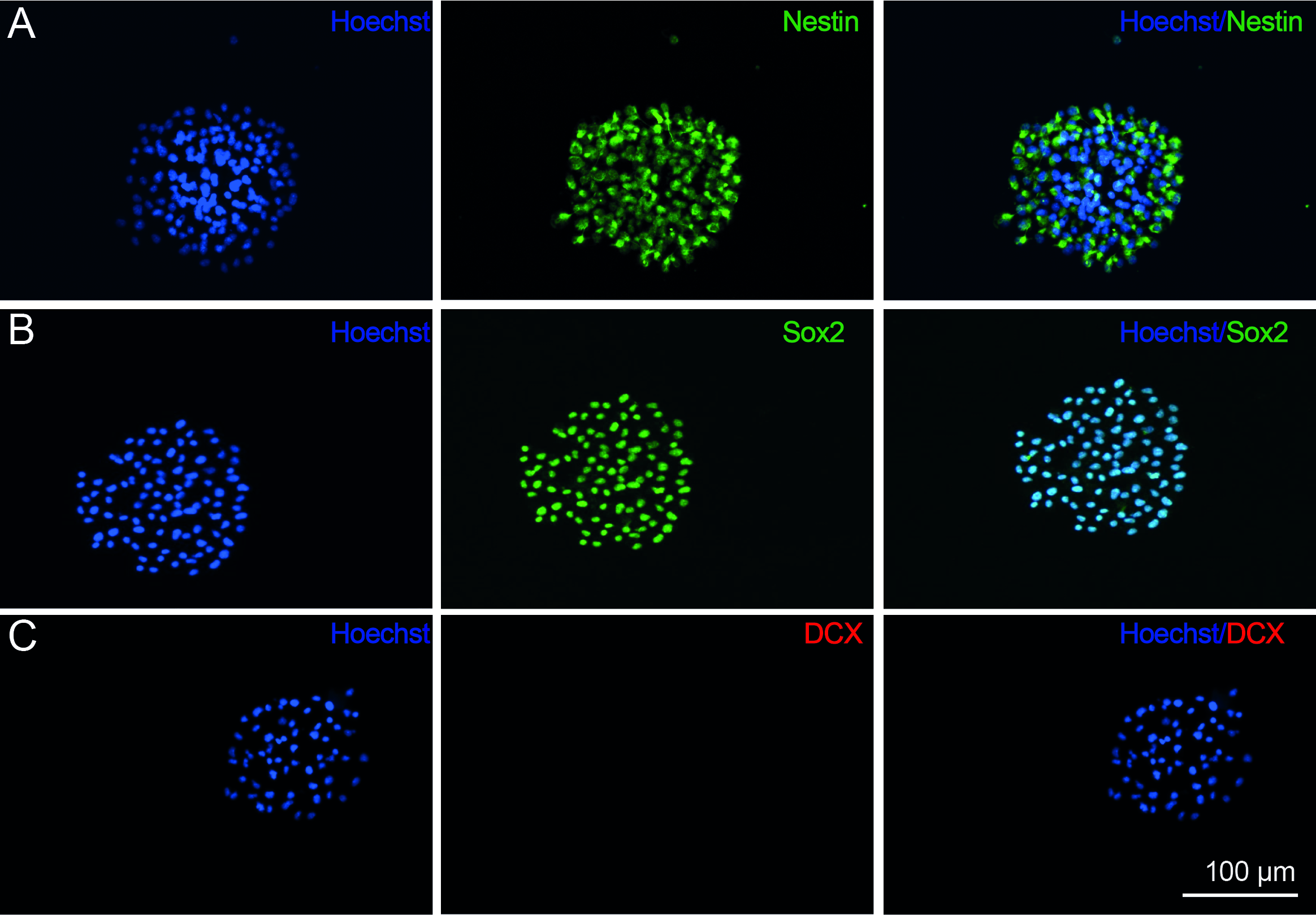

Supplement: Supplementary file 2 — Supplementary file2 (TIF 7172 KB)—Expression of NPC markers in NPC- neurospheres.Representative immunofluorescence microphotographs of secondary neurospheres before differentiation. The nuclei were stained with Hoescht (blue signal). Neurospheres were immunopositive to A) nestin and B) SOX-2. C) Neurospheres did not exhibit immunopositivity for DCX. Microphotographs were taken with a 20x magnification lens. The scale bar applies to all panels [file 11064_2023_3883_MOESM2_ESM.tif]

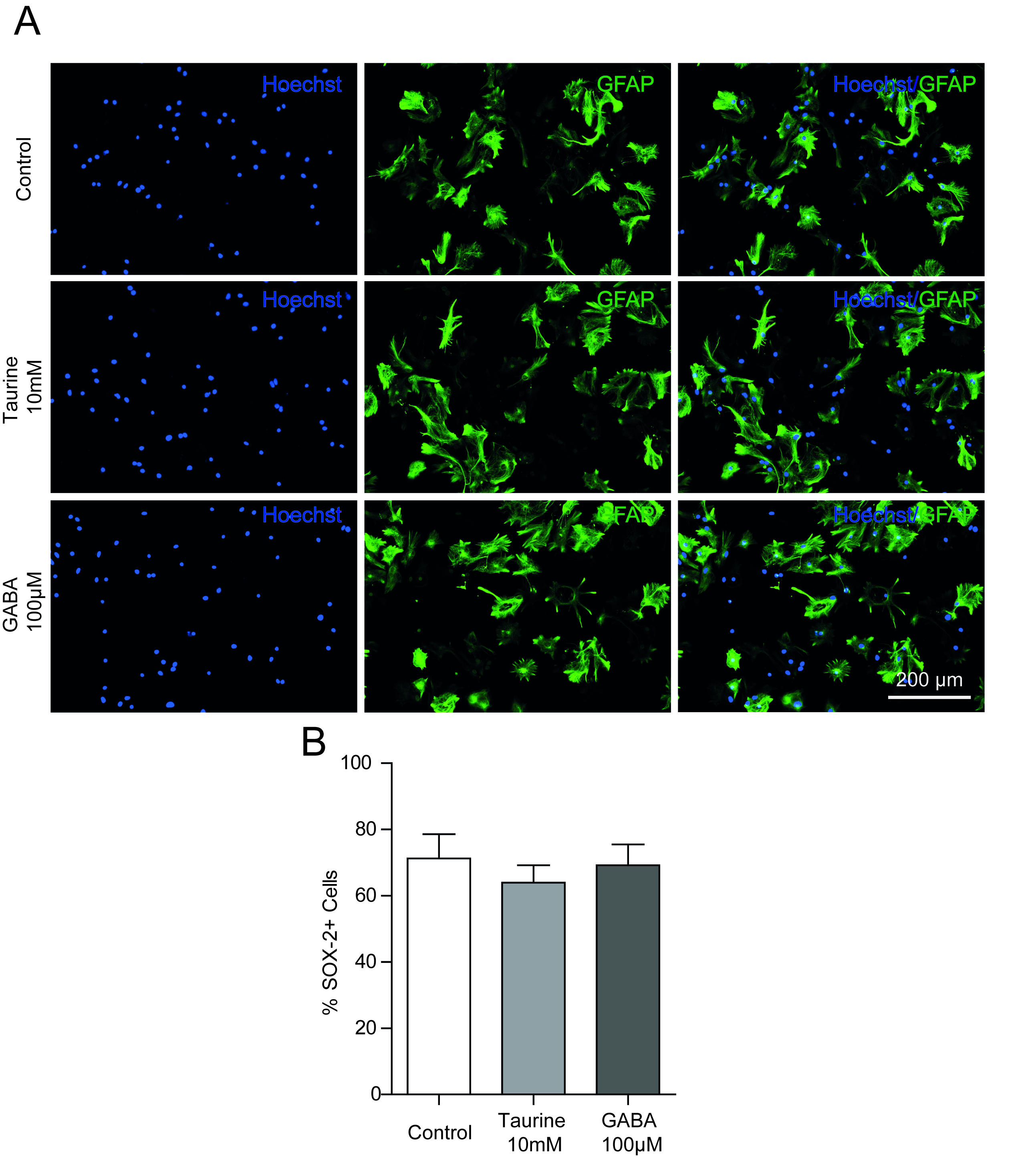

Supplement: Supplementary file 3 — Supplementary file3 (TIF 34018 KB)—Effect of taurine and GABA on the GFAP expression of disaggregated NPC-SVZ. A–C) Representative immunofluorescence microphotographs of disaggregated NPC-SVZ after the differentiation process in the three experimental conditions showing GFAP+ cells, including their characteristic morphology: abundant cytoplasm with elongated, stellate, or round shapes. The nuclei were stained with Hoescht (blue signal). The scale bar applies to all the microphotographs. B) Bar graph showing the percentage of GFAP+ cells in each experimental condition [file 11064_2023_3883_MOESM3_ESM.tif]
